# Deep learning-based automated detection of endometrioid endometrial carcinoma in histopathology

**DOI:** 10.3389/fonc.2025.1701427

**Published:** 2026-01-12

**Authors:** Ruotong Li, Kunyu Zou, Qihang Ma, Yaping Liu, Xiaohui Wang, Wenbin Huang, Shegan Gao, Xueying Yang

**Affiliations:** 1 The First Affiliated Hospital, and College of Clinical Medicine of Henan University of Science and Technology, Luoyang, China; 2 School of Computer and Communication Engineering, University of Science and Technology Beijing, Beijing, China; 3 College of Clinical Medicine, Henan University of Science and Technology, Luoyang, China; 4 School of Mechanical Engineering, University of Science and Technology Beijing, Beijing, China; 5 Department of Pathology, The First Affiliated Hospital of Henan University of Science and Technology, Luoyang, China; 6 Henan Key Laboratory of Microbiome and Esophageal Cancer Prevention and Treatment; Henan Key Laboratory of Cancer Epigenetics; Cancer Hospital, The First Affiliated Hospital (College of Clinical Medicine) of Henan University of Science and Technology, Luoyang, China; 7 Department of Gynecologic Oncology, The First Affiliated Hospital of Henan University of Science and Technology, Luoyang, China

**Keywords:** artificial intelligence, convolutional neural network, deep learning, endometrioid endometrial carcinoma, endometrium, pathology

## Abstract

**Background:**

Rapid advances in artificial intelligence (AI) have enabled automated tumor identification. To overcome challenges in traditional pathology, including complex sampling and limited physician resources, accessible tools for automated diagnosis are urgently needed.

**Methods:**

We developed a deep learning system based on an improved ResNet-18 to automatically identify endometrioid endometrial carcinoma (EEC) from H&E-stained endometrial hyperplastic lesions and normal tissues.

**Results:**

The model demonstrated strong performance in detecting endometrioid endometrial carcinoma. The positive predictive value (PPV), defined as the proportion of true disease cases among all positive diagnostic results, reached 95.13%, and the F1-score, defined as the harmonic mean of precision and recall, reached 0.95. The model achieved a PPV of 87.15% and an F1-score of 0.87 for typical hyperplasia, as well as a PPV of 79.88% and an F1-score of 0.74 for atypical hyperplasia, both meeting clinically acceptable thresholds. For normal endometrial physiological states, the PPVs were 91.75% (proliferative phase), 80.94% (secretory phase), and 80.88% (menopausal phase).

**Conclusion:**

This multi-task deep learning system provides stable and efficient support for automated EEC identification and effectively classifies endometrial pathological and physiological states, demonstrating strong potential for clinical translation.

## 1 Introduction

Endometrial carcinoma (EC), one of the three major malignancies of the female reproductive system, has surpassed cervical cancer as the most prevalent gynecological cancer in Europe and North America, and its incidence has been rapidly increasing in China in recent years (1). Among the histological subtypes of EC, endometrioid endometrial carcinoma (EEC) predominates, accounting for approximately 70%–80% of cases. EEC has well-defined morphological features, including crowded “back-to-back” glands with smooth internal contours, lined by monolayer or pseudostratified columnar cells that maintain nuclear polarity. Its pathological images provide abundant quantifiable information (such as gland density, arrangement, nuclear size, and mitotic figures), which facilitates diagnosis and classification. From a prognostic perspective, compared with other types of endometrial carcinoma, EEC generally shows a better therapeutic response, with an overall 5-year survival rate of 60%-70%. In particular, among patients with FIGO stage I disease (2), the 5-year survival rate can exceed 90%. These characteristics highlight the critical importance of early diagnosis of EEC for improving patient survival, guiding clinical treatment, and postoperative follow-up management.

With the continuous improvement of computing power and the advancement of big data technologies, artificial intelligence (AI) has been increasingly integrated into multiple aspects of oncology with unprecedented depth and breadth. Among its subfields, deep learning (DL) plays a particularly significant role in tumor prediction, medical image analysis, drug development, treatment planning, and clinical decision support. Deep learning algorithms are widely used for the automated detection and segmentation of lesions such as pulmonary nodules, breast masses, and colon polyps (3). In numerous studies, their recognition sensitivity and diagnostic accuracy have matched or even surpassed those of experienced radiologists and pathologists. In tumor risk prediction and prognosis assessment, AI can integrate patients’ genetic information, clinical characteristics, and lifestyle data to construct personalized risk models and predict treatment responses, recurrence probabilities, and survival outcomes (4). In epidemiology and public health, AI is used to analyze regional cancer incidence and mortality trends, optimize screening strategies for high-risk populations, and simulate the allocation of cancer prevention and treatment resources. At the clinical level, AI-based decision support systems assist physicians in developing individualized surgical, radiotherapy, and chemotherapy plans, thereby enhancing the standardization and precision of cancer treatment. In processing scientific literature and electronic medical records, AI can automatically extract key information from pathological reports and identify tumor staging and molecular subtype characteristics, significantly improving the efficiency of data organization and evidence retrieval in clinical research.

Histopathological examination remains the gold standard for the diagnosis and grading of cancer in clinical practice. Its diagnostic accuracy has a direct impact on clinical staging, treatment planning, and prognosis assessment. However, the pathology workforce in China faces a dual challenge. First, there are only about 10,000 practicing pathologists, resulting in a shortage of nearly 90,000 compared with the WHO recommendation of 1–2 pathologists per 100 hospital beds. Second, the training of pathologists is lengthy, requiring 5 years of medical education, 3 years of standardized residency training, and 2 additional years of subspecialty training (5). Traditional diagnostic models depend on morphological interpretation by physicians using optical microscopes. This approach is time-consuming (averaging 30–45 minutes per case) and subject to inter-observer variability, with studies reporting diagnostic discrepancies among pathologists with different levels of experience (6).

The shortage of pathologists and diagnostic complexity constitute a significant challenge. Breakthroughs in AI technology provide a new paradigm for advancing pathological diagnosis. Deep learning-based whole-slide image (WSI) analysis has demonstrated significant advantages: For example, an algorithm developed by Google Health achieved an AUC of 0.993 in breast cancer sentinel lymph node detection (7). A study published in Nature Medicine demonstrated that an AI model could predict microsatellite instability status in colorectal cancer with 84% accuracy by extracting features from H&E-stained sections (8). These advances suggest that deep learning technology has the potential to overcome multiple limitations of traditional pathological diagnosis, enhancing efficiency while reducing human error.

AI technology is being widely applied to EC precise identification, risk stratification, and personalized treatment. In imaging, AI analysis of MRI, ultrasound, and other imaging data enables precise segmentation of uterine regions, assessment of tumor volume and infiltration extent, and even prediction of lymph node metastasis risk, thereby assisting FIGO staging and surgical decision-making. In pathological diagnosis, AI can efficiently analyze digitized WSIs of endometrial cancer, assisting pathologists in identifying subtle histological features, improving diagnostic accuracy for complex subtypes (e.g., serous carcinoma, clear cell carcinoma), and potentially quantifying prognostic indicators such as tumor-infiltrating lymphocytes (TILs) (9). In early endometrial cancer screening, AI models can achieve high-sensitivity identification of potential lesions through integrated analysis of clinical variables such as patient symptoms, endometrial thickness, and reproductive history, providing new technical support for early intervention, precise diagnosis, and intelligent management (10).

Although AI applications in endometrial cancer have achieved promising initial results, most studies focus on the overall diagnosis of endometrial cancer or the differentiation of malignant subtypes (e.g. serous carcinoma). There remains insufficient attention to the automation of diagnosis for EEC–the most clinically relevant subtype-and its closely related, critically significant precursor lesions (typical and atypical hyperplasia), as well as endometrial physiological staging. Such precise auxiliary diagnosis is crucial for guiding early treatment choices and prognosis assessment in EEC (11, 12). Therefore, this study aims to develop and validate a deep learning-based auxiliary diagnostic system for endometrioid endometrial carcinoma. This system utilizes routinely H&E-stained whole-slide images (WSIs) to accurately distinguish EEC from endometrial hyperplastic lesions (typical hyperplasia, atypical hyperplasia) and three key physiological states of the endometrium (proliferative phase, secretory phase, menopausal phase). It is anticipated that this auxiliary diagnostic system will provide a clinically feasible solution to address the shortage of pathological resources while improving diagnostic efficiency and consistency.

## 2 Materials and methods

### 2.1 Data collection

In this study, we aimed to develop an intelligent recognition model for high-precision automated identification of endometrioid endometrial carcinoma. The research data were obtained from archived high-quality samples in the Department of Pathology, The First Affiliated Hospital of Henan University of Science and Technology (13, 14). Data collection followed a retrospective study design. We systematically screened endometrial pathological slides prepared at this hospital between 2019 and 2024. Before inclusion in the analysis, all slides underwent rigorous quality review to ensure suitability for subsequent digitization and model training. The study included only classic Hematoxylin and Eosin (H&E)-stained slides, which are the most fundamental and widely used technique in pathological diagnosis (15–18). A total of 767 pathological slides were collected, including EEC (551 slides), secretory phase (49 slides), menopausal phase (41 slides), proliferative phase (42 slides), typical hyperplasia (50 slides), and atypical hyperplasia (34 slides) (**Table 1**). To ensure the reliability and validity of the diagnostic labels, all 767 slides were independently reviewed by two senior pathologists from the same institution. In cases of diagnostic disagreement, consensus was reached through joint discussion, thereby confirming the category of each slide and ensuring the consistency and accuracy of the dataset.

**Table 1:** Distribution of pathological categories in training and testing sets.

Category	Training samples	Testing samples	Total samples
Endometrioid endometrial carcinoma	324	227	551
Atypical Hyperplasia	24	10	34
Typical Hyperplasia	35	15	50
Proliferative phase	29	13	42
Secretory phase	34	15	49
Menopausal phase	28	13	41
Total	474	293	767

### 2.2 Data preprocessing

**Figure 1:**
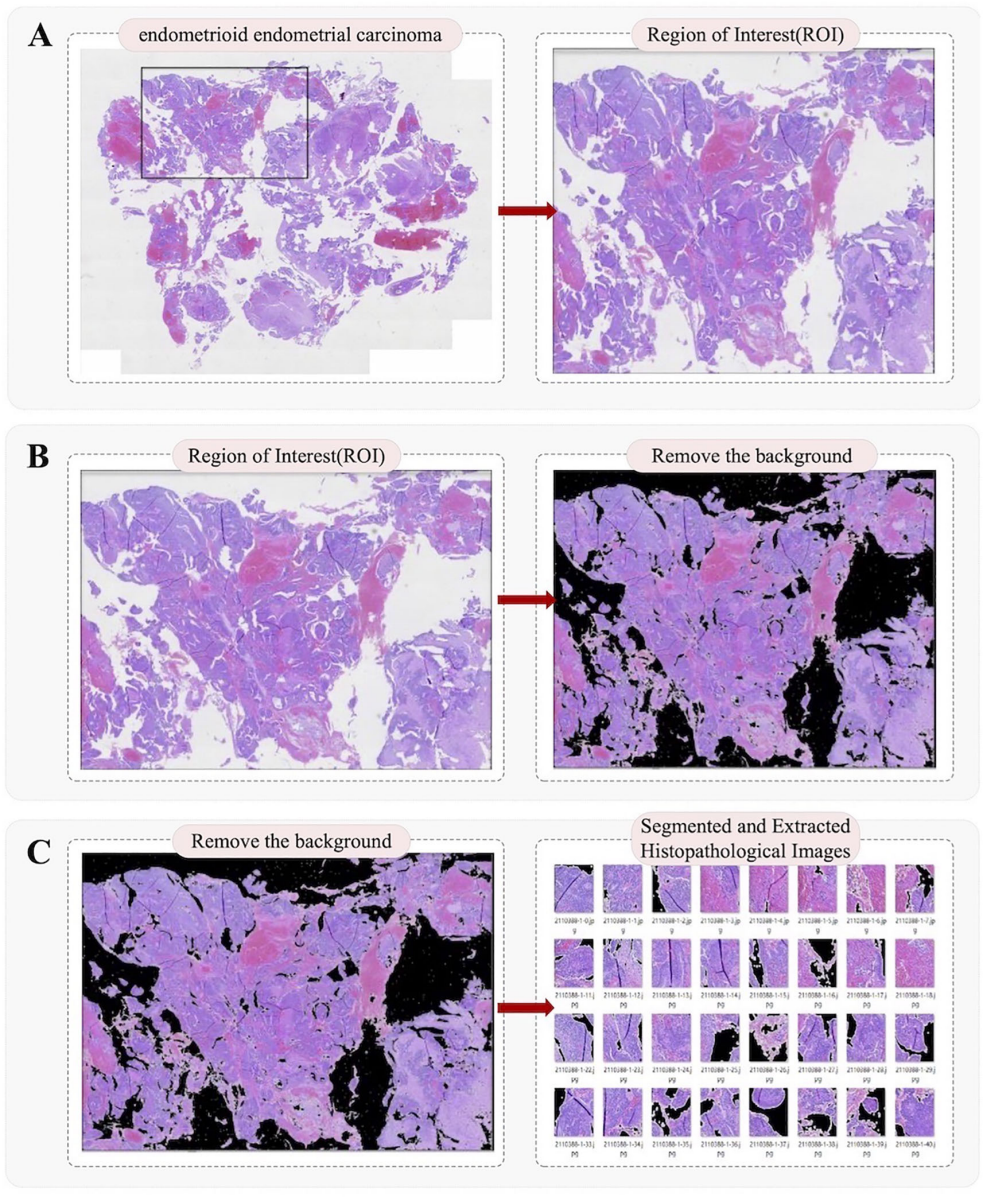
Endometrial pathological image preprocessing workflow. (**A)** WSI scanning and region annotation: H&E-stained whole slide scanned at 20× magnification; target regions outlined according to WHO standards. **(B)** Extraction of annotated regions: Outlined regions undergo grayscale conversion, maxima suppression, dilation, and erosion operations to generate tissue masks. **(C)** Sample generation: A sliding window (256 × 256 pixels) samples patches from the tissue mask.

**Figure 2:**
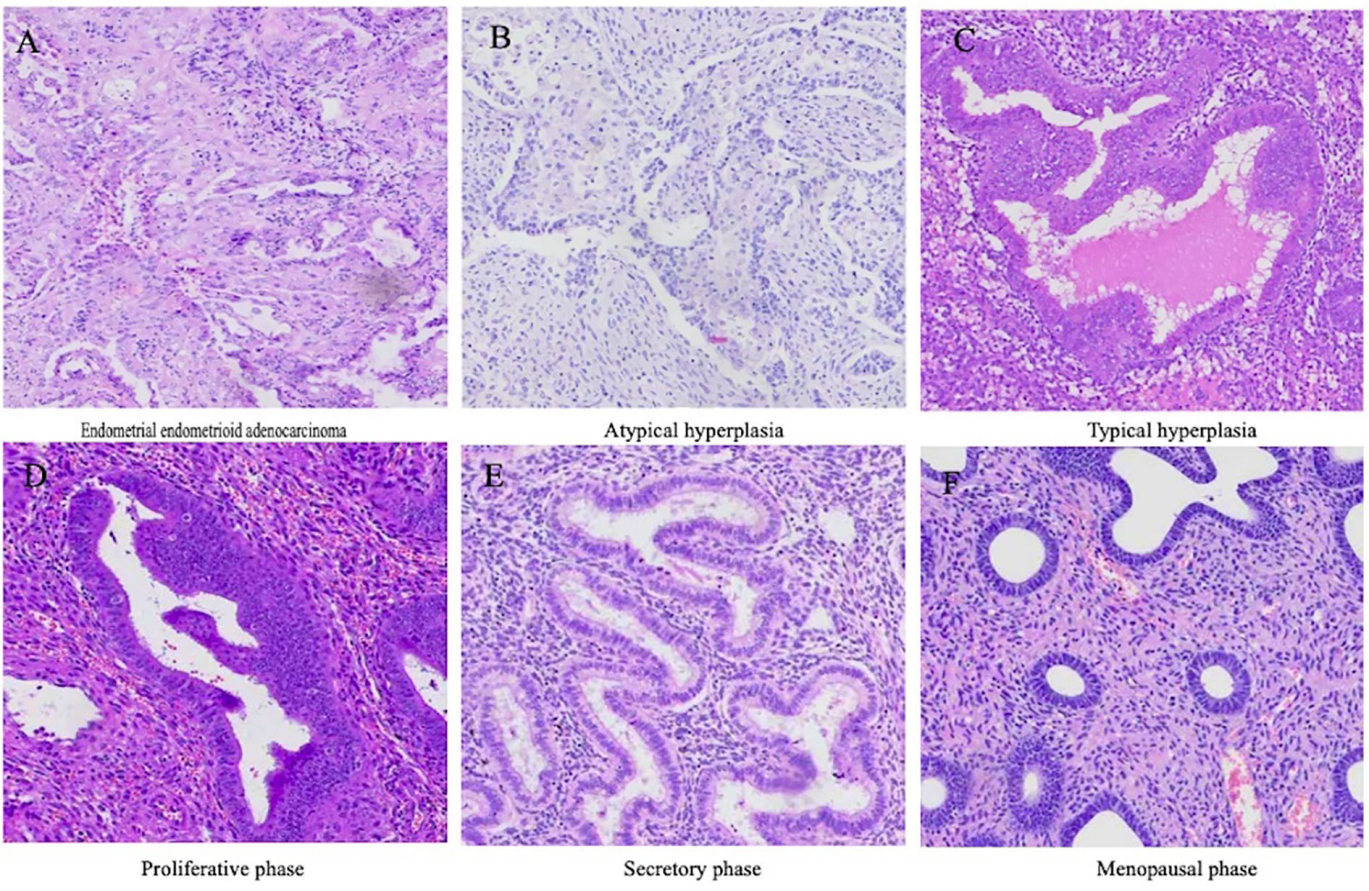
Representative histological images for the six-class endometrial classification task in this study. The figure illustrates the six endometrial categories targeted for differentiation in this study; **(A)** Endometrioid endometrial carcinoma(EEC); **(B)** Atypical hyperplasia; **(C)** Typical hyperplasia; **(D)** Proliferative phase; **(E)** Secretory phase; **(F)** menopausal phase. These images highlight the distinct morphological characteristics of each category and serve as the foundation for model training and evaluation. All images are stained with hematoxylin and eosin (H&E).

First, all whole-slide images (WSIs) were scanned at high quality using a Motic Easyscan scanner, with the objective magnification fixed at 20× to ensure image clarity and structural integrity (19, 20). The digitized WSIs were exported from the scanner. During export, image resolution and quality were checked to meet the requirements for subsequent analysis, ensuring suitability for annotation and processing (21). The exported images were stored in a dedicated database and categorized by patient ID and slide type to facilitate retrieval and management. During the annotation phase, the WSIs were annotated using in-house software developed on MacOS, following World Health Organization (WHO) standards for region delineation (**Figure 1**). Based on pathological features, different areas within each slide were delineated, including normal endometrium, hyperplastic regions, and cancerous regions. Polygons or rectangular boxes were drawn using software tools to precisely mark the boundaries of each region and add annotations (**Figure 2**). After initial annotation, all WSIs were reviewed by two senior pathologists for verification. They assessed the accuracy and consistency of each region and further examined whether the annotation boundaries were appropriate and whether the feature descriptions complied with pathological standards. Following expert verification and correction, all annotation results were finalized and used for model training.

## 3 Proposed method

### 3.1 Sample preparation

**Figure 3:**
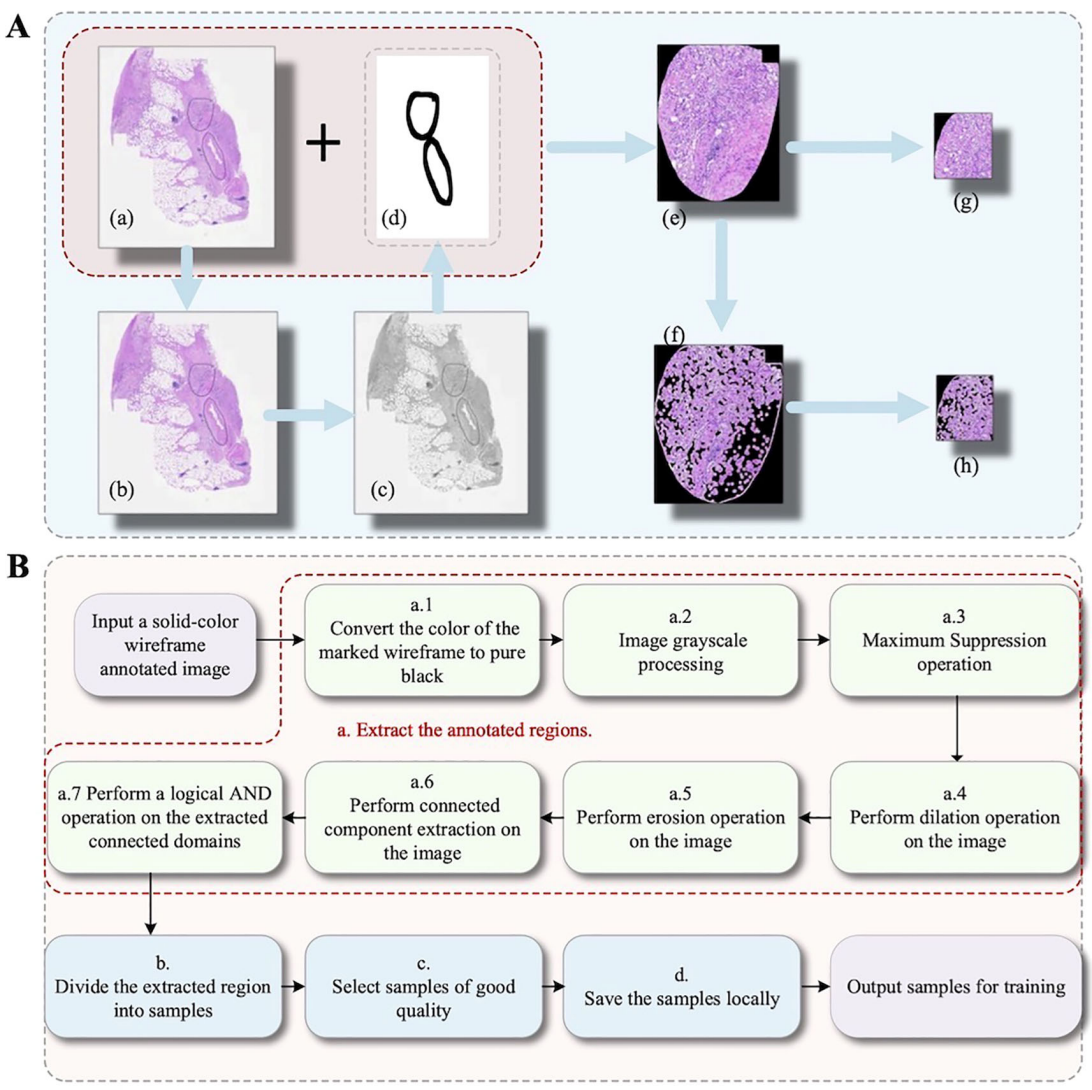
Sample generation flowchart and output results. **(A)** Sample generation: a: Extract marked regions; b: Segment samples; c: Sample optimization (selecting high-quality samples); d: Sample saving. **(B)** Output image results for each step.

Existing pathological image analysis techniques primarily rely on manual annotations or conventional image processing methods, such as thresholding, connected component extraction, and morphological operations, to identify regions of interest in tissue slides. Building on these techniques, we developed a systematic pipeline to generate high-quality training samples for deep learning. We adopted a three-step approach for classification. In the initial step, the annotated regions were first extracted. The annotations used were solid-colored, irregular, nearly closed line contours. The areas inside these contours were the cell images requiring recognition. The image format used was JPG; non-JPG formats were first converted to JPG. Next, the solid color used for annotation was converted to pure black (RGB (0, 0, 0):). The image with converted annotation color was then grayscaled (22). Grayscale images underwent maximum suppression, where all pixel values greater than 0 were set to 255. Using the cv2.connectedComponentsWithStats(thresh, connectivity, cv2.CV_32S) function from the OpenCV library (23), with dilate_iterations set to 1, dilation was performed on the suppressed black regions. Using the cv2.erode(thresh, kernel, iterations=erode_iterations) function from OpenCV, erosion was performed on the dilated image. After processing, connected component extraction was performed on the image using OpenCV’s cv2.connectedComponentsWithStats(thresh, connectivity, cv2.CV_32S) with connectivity set to 4. The resulting connected regions represented the annotated areas (24). A list of masks for the annotated regions was then obtained. The masks were intersected with the original image to extract the annotated regions. The extracted annotated regions were traversed, and for each annotated region, a filtering operation was performed to obtain filtered images. For both the original annotated regions and the filtered annotated regions, image patches were extracted using a sliding window approach starting from the pixel at position (0,0). The window size was 256 pixels × 256 pixels, with a stride of 256 pixels in both width and length directions (25, 26). Each sampling position generated a pair of samples: one from the annotated region (pi) and the other from the filtered annotated region (pi′). We used the third-party library histolab.filters.image_filter with four functions: RgbToGrayscale, OtsuThreshold, BinaryDilation, and ApplyMaskImage. This allowed us to optimize tissue regions, reduce background interference, and ultimately obtain higher-quality training samples (**Figure 3**).

### 3.2 Model construction and training

Considering ResNet-18’s relatively lightweight structure, strong generalization ability, and suitability for extracting multi-level features from limited pathological samples, we chose it as the base deep learning mode. During the data loading stage, we used the Dataset and DataLoader modules from the PyTorch deep learning framework to read the selected samples. First, samples from all six pathological categories were divided into training and test sets in a 7:3 ratio (27). Specifically, the training set contained 70% of the samples, while the test set contained 30% of the positive samples and the remaining samples from the other five categories. Corresponding sample label lists were generated by assigning labels based on the training sample order. In this study, the six categories were first assigned integer codes 0-5, and later converted to one-hot encoding during processing. To enhance model generalization, the order of samples in the training set was randomly shuffled to prevent model dependence on sample sequence. During model parameter setting, we selected ResNet18 as our deep learning model. ResNet consists of 18 layers, including 16 convolutional layers and two fully connected layers (28, 29), primarily characterized by strong computational capability and relatively low computational complexity. To leverage prior knowledge, we pre-loaded the official PyTorch-provided pre-trained weights before training. As the task involved six-class classification, we modified the final output layer of ResNet-18, adjusting its output dimension to 6. This modification ensured the model could adapt to our specific task requirements. During training parameter setting, several key parameters were configured to optimize the training process. Specifically, the batch size was set to 16, the num_workers parameter was set to 0 to ensure stability and efficiency in data loading. Training was performed using a GPU to accelerate computation (30). The objective function chosen for training was the Cross Entropy Loss, suitable for multi-class classification problems and effective in measuring the difference between model output and true labels. For optimization, the Stochastic Gradient Descent (SGD) algorithm was used with an initial learning rate of 0.001 to achieve faster convergence in the early training stages. The model learned the discriminative features of different pathological classes through end-to-end learning.

### 3.3 Inference process

**Figure 4:**
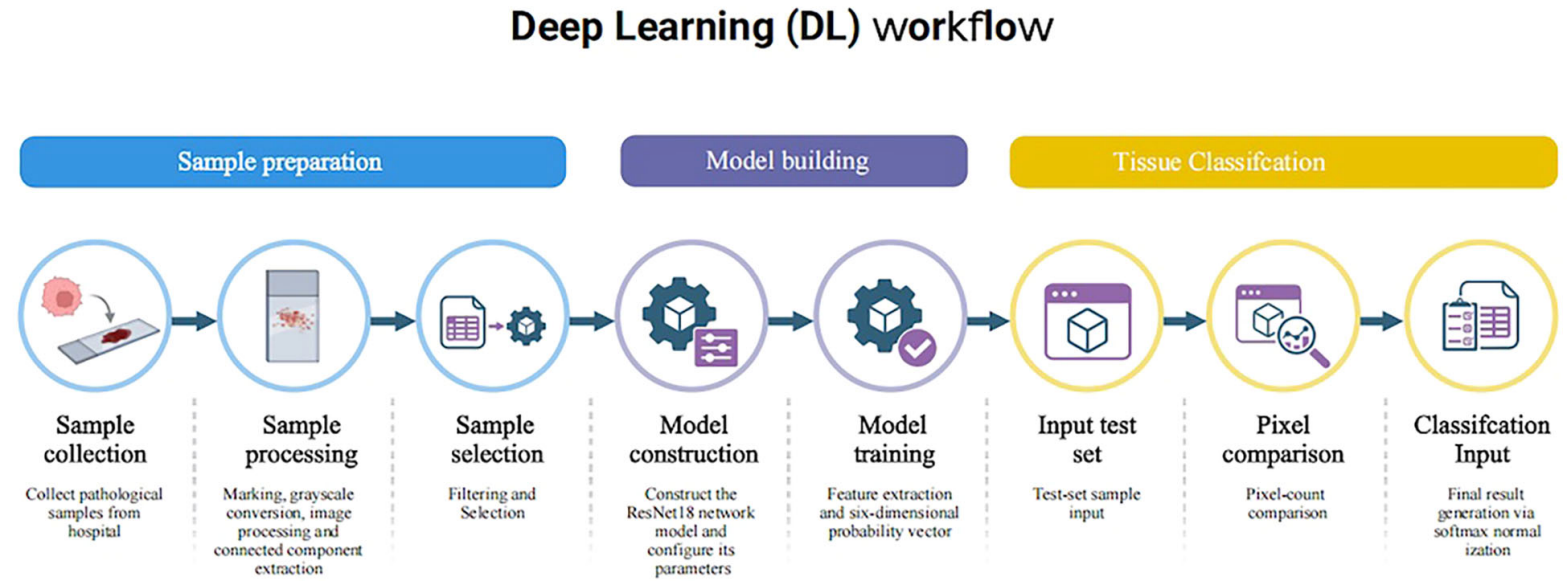
Workflow of the deep learning (DL) framework. The workflow begins with sample preparation, including the collection, processing, and selection of pathological samples; next, the ResNet-18 network model is constructed and trained; finally, the results for the test set samples are produced.

For the pathology images to be tested, the same filtering operation was performed. The sliding window intercepted the samples qi and qi′. The number of black and white pixels in qi′ was counted. If the number of black pixels exceeded half of the total number of pixels, the corresponding location in the original image, qi, was recorded as an unorganized region. If the number of black pixels was less than half of the total number of pixels, qi was input into the trained six-class classification model. The model output was normalized using `Softmax`, generating six-dimensional probability vectors corresponding to the confidence levels of the six pathology categories. Finally, the results from all windows were integrated to generate the probability distribution of pathological categories for the entire image (**Figure 4**).

### 3.4 Primary outcome measures and statistical methods

The performance of the deep learning model in multi-class classification tasks was systematically evaluated using confusion matrix heatmaps and Receiver Operating Characteristic (ROC) curves (31–33). Core evaluation metrics included sensitivity, specificity, Positive Predictive Value (PPV), Negative Predictive Value (NPV), F1-score (the harmonic mean of precision and recall) (**Table 2**), and Area Under the ROC Curve (AUC), providing a comprehensive assessment of the model’s classification accuracy, stability, and clinical utility. Continuous variables were expressed as mean ± standard deviation, and categorical variables as percentages with their 95% confidence intervals (95% CI). Since the primary objective of this study was to assess the performance of a single deep learning model on an independent test set—rather than to compare different models or sample groups—no between-group hypothesis testing was required. Statistical inference was instead based on 95% confidence intervals derived from binomial distribution assumptions, which were used to quantify the uncertainty of each performance metric. This method ensured statistical rigor and avoided introducing comparative tests that were not aligned with the study design. To ensure rigor and objectivity, all evaluations were performed on an independent test set. This approach ensured that the results reflected the model’s generalization ability and provided a basis for assessing its feasibility in clinical applications.

**Table 2:** Evaluation metrics, formulas, and interpretations for multi-class classification of pathological images.

Metric	Formula	Interpretation
Sensitivity (Recall)	Sensitivity = TP/(TP + FN)	Ability of the model to correctly identify positive samples
Specificity	Specificity = TN/(TN + FP)	Ability of the model to correctly identify negative samples
Positive Predictive Value (PPV/Precision)	Precision = TP/(TP + FP)	Proportion of predicted positives that are true positives
Negative Predictive Value (NPV)	NPV=TN/(FN+TN)	Proportion of predicted negatives that are true negatives
F1-Score	F1 = 2 × (Precision × Recall)/(Precision + Recall)	Harmonic mean of precision and recall, balancing accuracy and recall

## 4 Results

### 4.1 Pathological identification results of endometrioid endometrial carcinoma

**Figure 5:**
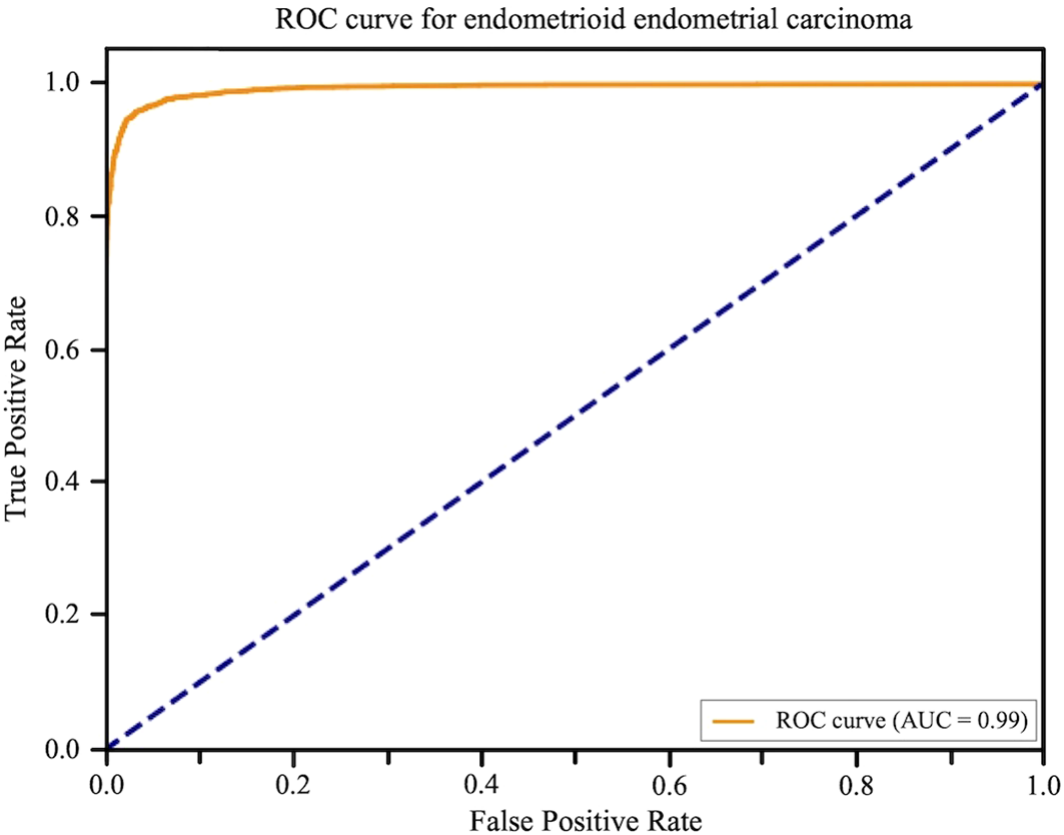
Receiver operating characteristic (ROC) curve for the endometrioid endometrial carcinoma identification model. ROC curve based on the internal test set demonstrates excellent performance: Area Under Curve (AUC) = 0.99, Sensitivity 96.06%, PPV 95.13%.

**Figure 6:**
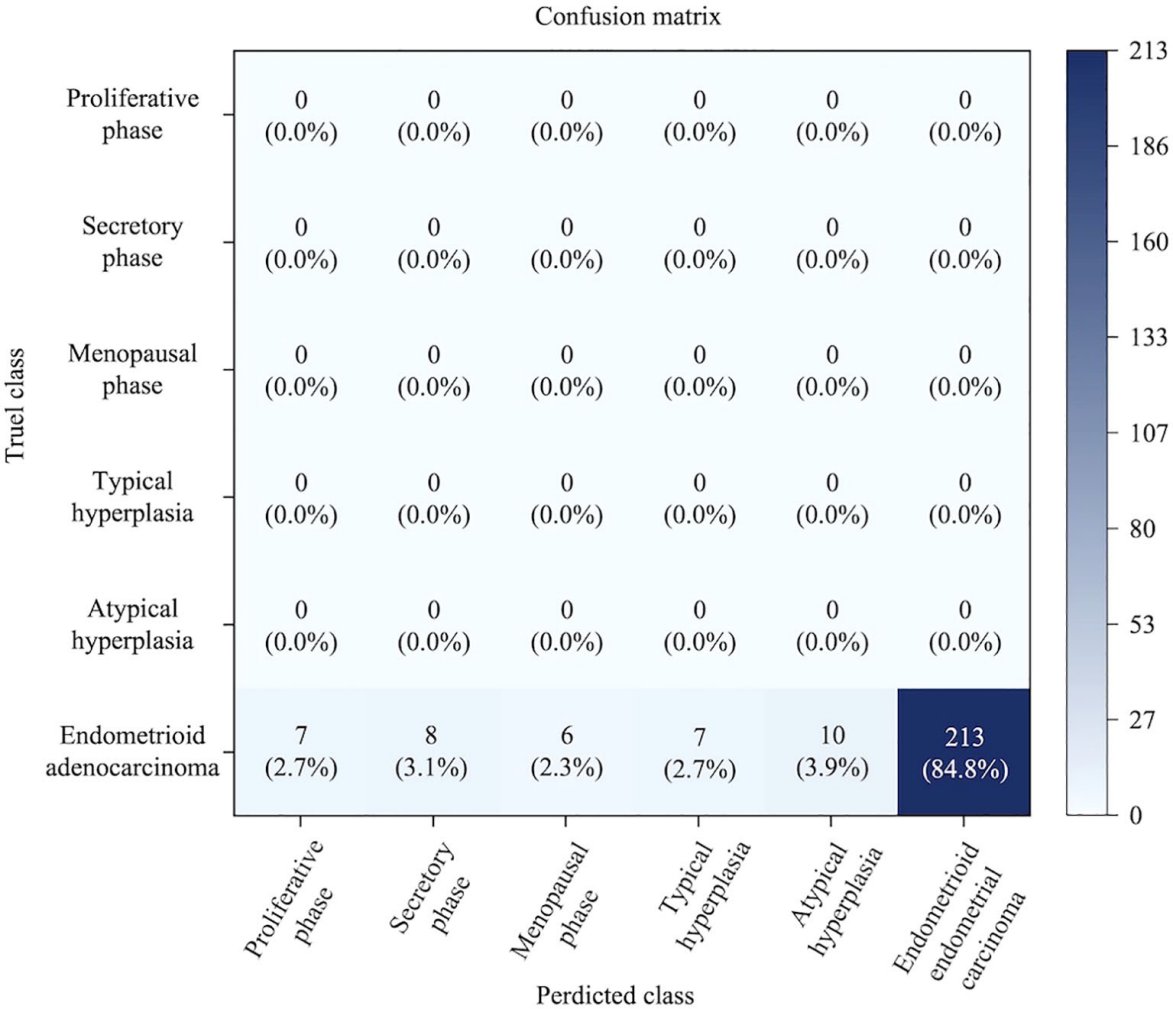
Performance evaluation on the independent validation set. The model achieved 84.86% accuracy in identifying endometrioid adenocarcinoma on the external dataset, showing a moderate decline compared to the internal test set.

In the dataset partition, from a total of 551 patients with EEC, pathological slides from 463 patients were randomly selected for model development (including training and validation). The slides were randomly divided: approximately 70% (324 cases) were used for model training, enabling the model to learn the complex morphological features of EEC. The remaining 30% (139 cases), together with images from other categories, formed an internal test set, which was used to evaluate recognition accuracy and model robustness after development. After training and optimization, the model demonstrated excellent performance on the internal test set, achieving a sensitivity of 96.06% and a PPV of 95.13%, successfully distinguishing cancerous tissue from other categories (**Figure 5**). This indicates that after learning from a large number of representative samples, the model acquired strong ability to accurately differentiate endometrial carcinoma tissue from other categories encountered during training (such as normal phases and hyperplasia), marking initial success in the core task of automated identification of EEC and laying the foundation for broader validation. To further assess the model’s generalization ability and potential clinical value, the 463 samples used for development (training and internal testing) were excluded, and slides from the remaining 88 patients with EEC were reserved as a completely independent validation set. These slides were entirely unseen during model training and optimization, providing a more rigorous and realistic testing environment. The model achieved an accuracy of 84.86% on this independent validation set (**Figure 6**). Although slightly lower than that of the internal test set, this result still reflects the strong potential of deep learning technology in pathological image analysis and its ability to provide reliable support for endometrioid endometrial carcinoma.

### 4.2 Pathological identification results of endometrial hyperplasia

**Figure 7:**
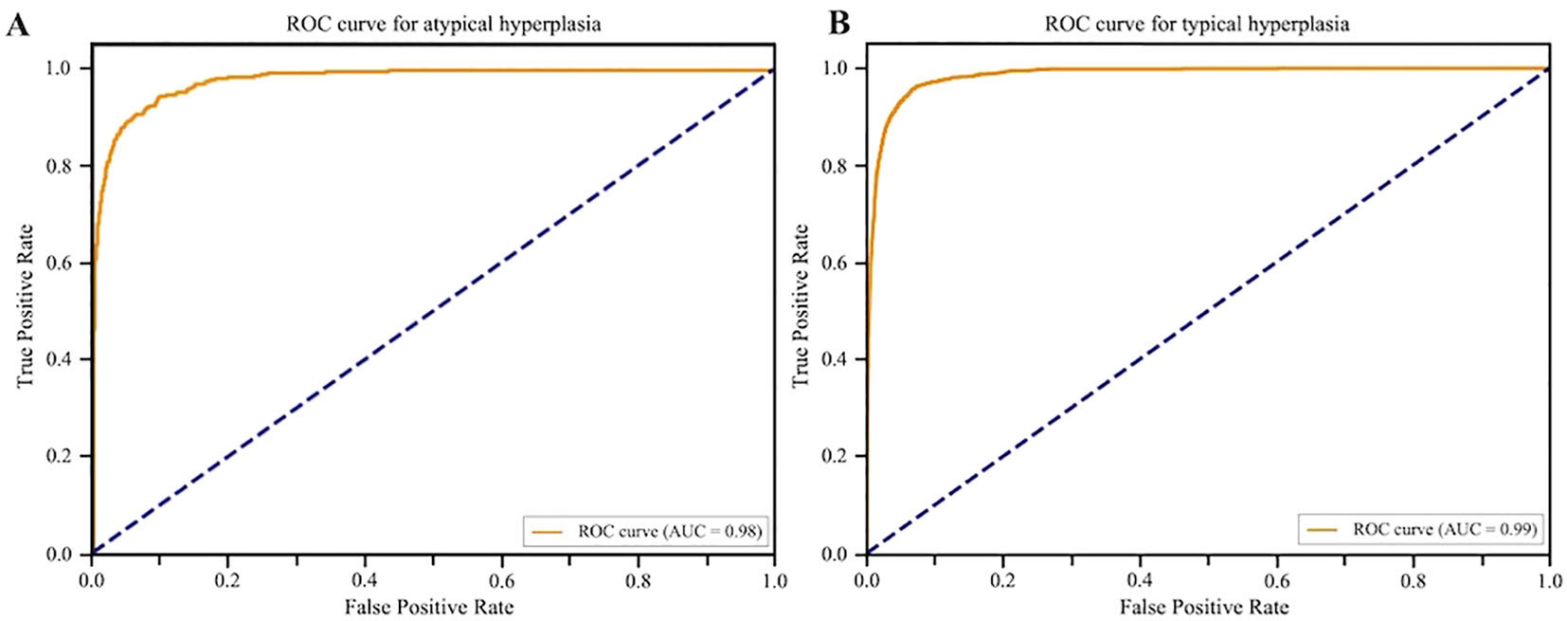
Receiver operating characteristic (ROC) curves for endometrial hyperplasia subtypes. **(A)** Atypical Hyperplasia, ROC curve (AUC = 0.98), Internal test set performance: PPV 79.88%, F1 score 0.74. **(B)** Typical Hyperplasia, ROC curve (AUC = 0.99), Internal test set performance: PPV 87.15%, F1 score 0.87.

Building on the accurate identification of EEC, the recognition of endometrial hyperplasia facilitates the detection of precursor lesions and supports early intervention and cancer risk assessment (34). In the intelligent diagnosis research for endometrial lesions, the accurate discrimination of hyperplastic lesions holds significant clinical importance. We particularly focused on the model’s performance in identifying two key lesions: Typical Hyperplasia and Atypical Hyperplasia, which exhibit significant differences in cytological atypia, structure, and arrangement pathologically. To ensure rigorous evaluation, we employed a stratified data partitioning strategy consistent with the core model development: From a total of 84 hyperplasia samples (50 typical hyperplasia, 34 atypical hyperplasia), 70% of samples (35 typical hyperplasia, 24 atypical hyperplasia) were randomly selected for the training set to enable the model to learn the morphological features of hyperplastic lesions. Simultaneously, the remaining 30% of samples along with samples from other categories constituted the independent test set to objectively assess the model’s ability to recognize hyperplasia samples. After systematic training and optimization, the deep learning model achieved clinically acceptable standards on the independent test set: Typical Hyperplasia (PPV 87.15%, F1-score 0.87) (**Figure 7**). For Atypical Hyperplasia, which presents greater structural atypia and diagnostic challenges, the model also demonstrated a certain level of discriminative capability (PPV 79.88%, F1-score 0.74). This fully indicates that the model is not only suitable for detecting malignant lesions (endometrial carcinoma) but also possesses excellent capabilities in the fine-grained classification of hyperplastic lesions. This suggests that the model achieves more comprehensive coverage of endometrial pathologies.

### 4.3 Pathological identification results of endometrial physiological stages

**Figure 8:**
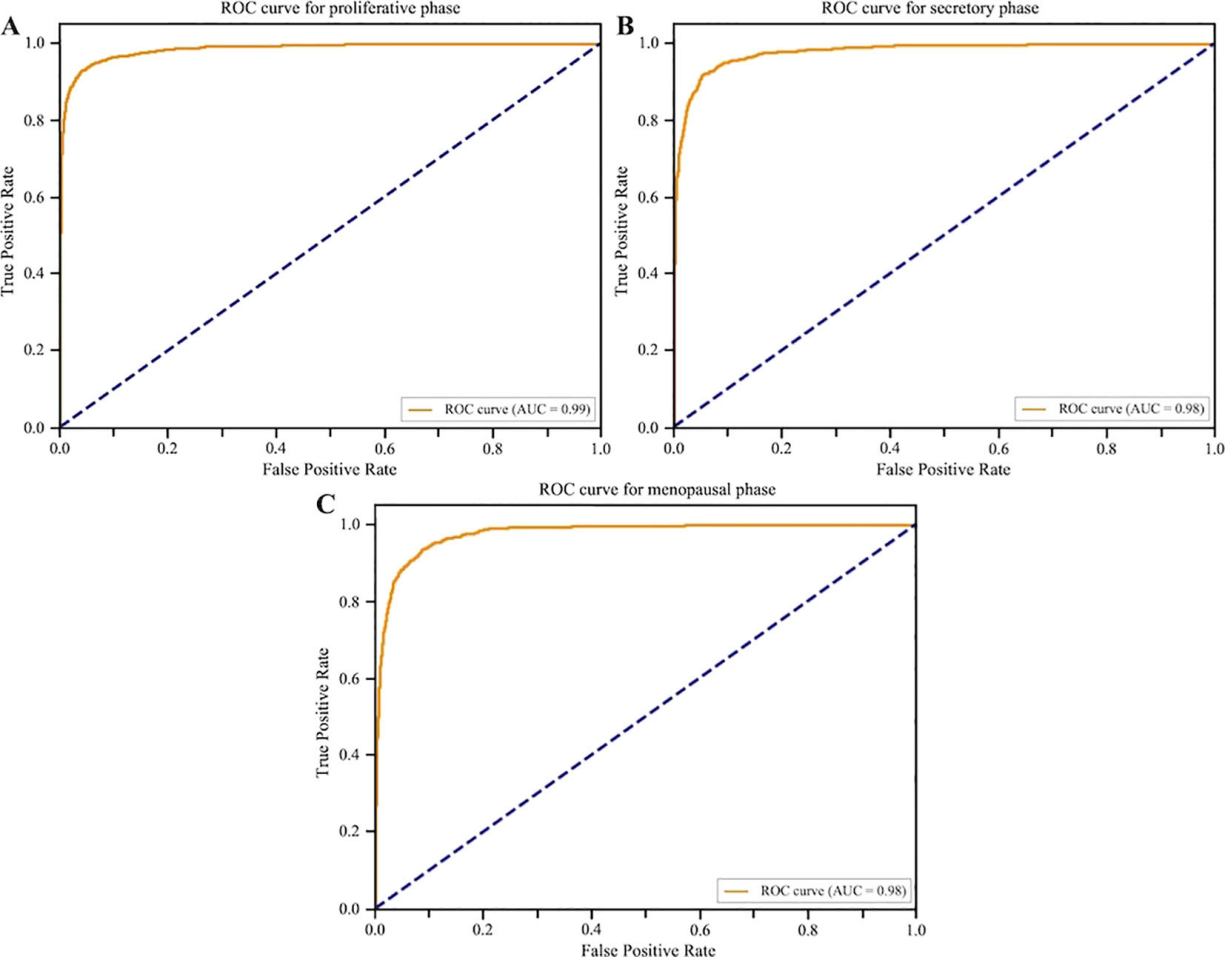
Receiver operating characteristic (ROC) curves for normal endometrial phases. **(A)** Proliferative Phase, ROC curve (AUC = 0.99), Internal test set performance: PPV 91.75%, F1 score 0.88. **(B)** Secretory Phase: ROC curve (AUC = 0.98), Internal test set performance: PPV 80.94%, F1 score 0.81. **(C)** Menopausal Phase: ROC curve (AUC = 0.98), Internal test set performance: PPV 80.88%, F1 score 0.77.

Accurate identification of normal endometrial physiological phases is critical for distinguishing pathological changes from physiological variations, thereby enhancing the reliability of EEC diagnosis. Targeting three key physiological stages of clear clinical significance (the Proliferative Phase, Secretory Phase, and Menopausal Phase), we employed a stratified data partitioning strategy consistent with the overall study: A total of 132 physiological phase samples (42 proliferative, 49 secretory, 41 menopausal) were divided in a stratified random 7:3 ratio. 70% of the samples were used to train the model to capture phase-specific microscopic features (e.g. gland morphology, stromal cell density, vascular distribution, epithelial height changes). The remaining 30% of samples, along with samples from other categories, formed the independent test set to evaluate model generalization. The model achieved high accuracy but also showed significant variation across the highly similar normal physiological phases on the independent test set: Proliferative Phase (PPV 91.75%), Secretory Phase (PPV 80.94%), and Menopausal Phase (PPV 80.88%; **Figure 8**). Minor misclassifications occurred primarily between the secretory and proliferative phases. This misclassification aligns precisely with challenging points in traditional pathological diagnosis, demonstrating that the deep learning model can automate the identification of key endometrial physiological phases (**Table 3**).

**Table 3:** Performance metrics of the deep learning model for six-class classification task.

Category	Sensitivity (%)	Specificity (%)	PPV (%)	NPV (%)	F1 Score	AUC (95% CI)
Endometrioid endometrial carcinoma	96.06	96.14	95.13	96.88	0.95	0.99
Atypical Hyperplasia	70.20	99.11	79.88	98.52	0.74	0.98
Typical Hyperplasia	87.09	97.31	87.15	97.29	0.87	0.99
Proliferative phase	85.79	98.66	91.75	97.56	0.88	0.99
Secretory phase	81.41	97.81	80.94	97.88	0.81	0.98
Menopausal phase	73.83	98.28	80.88	97.44	0.77	0.98

To further clarify the actual performance and advantages of our model, we conducted a comparative analysis with existing methods. Our model achieved an F1-score of 0.95, a sensitivity of 96.06%, and PPV of 95.13% in the task of EEC identification, substantially outperforming the average performance of most current methods, which typically reach F1-scores of 0.85–0.90. In the classification of hyperplastic lesions, our model attained F1-scores of 0.87 and 0.74 for typical and atypical hyperplasia, respectively, demonstrating stronger recognition capabilities compared to existing approaches. Additionally, in identifying normal endometrial physiological phases, our model achieved PPVs of 91.75%, 80.94%, and 80.88% for the proliferative, secretory, and menopausal phases, respectively, accurately capturing the characteristics of each phase and aiding in distinguishing pathological changes from normal physiological stages. These results indicate that our model not only excels in EEC identification but also exhibits robust performance in recognizing other endometrial lesion types and physiological phases, highlighting its potential utility in clinical auxiliary diagnosis.

## 5 Discussion

With the rising incidence of endometrial carcinoma and the increasing number of biopsy samples (35, 36), manual pathological diagnosis faces an ever-growing workload and significant challenges. To overcome these limitations and improve diagnostic efficiency, this study developed a high-performance deep learning model that enables precise identification of endometrioid endometrial carcinoma, providing clinicians with a more effective and objective diagnostic aid.

AI technologies, particularly machine learning (ML) and deep learning algorithms, are profoundly advancing oncology research and clinical practice. Traditional ML methods, such as Support Vector Machine (SVM), Random Forest (RF), k-Nearest Neighbors (KNN), and ensemble learning models (e.g., XGBoost and LightGBM) (37–39), are widely applied to structured data analysis tasks including classification, feature selection, and prognostic model construction, owing to their strong interpretability and adaptability. In contrast, deep learning, driven by multi-layer neural networks, can automatically extract latent features from complex datasets, making it especially suitable for analyzing unstructured data such as medical images, histopathological slides, and time-series signals. Common deep learning architectures include Feedforward Neural Networks (FNNs), Convolutional Neural Networks (CNNs), Recurrent Neural Networks (RNNs), Residual Networks (ResNets), and the rapidly evolving Graph Neural Networks (GNNs) (40–42). Each type of algorithm demonstrates unique advantages across different data modalities, thereby greatly facilitating the development of precision medicine. In radiomics, CNNs have been extensively employed for automated tumor image segmentation and classification. Deep learning models exemplified by 3D U-Net achieve precise mapping from raw pixels to tumor voxel probabilities through mechanisms such as skip connections, Dice/Focal Loss to address class imbalance, and 3D spatial context modeling. In gene expression and multi-omics analysis, algorithms such as SVMs and RFs are commonly used for tumor subtype discrimination and survival prediction (43), offering both interpretability and generalizability. Furthermore, ensemble learning approaches, including XGBoost and LightGBM, are frequently applied in the construction of prognostic models and risk scoring systems. More recently, GNNs have been increasingly adopted to investigate cell-cell interactions within the tumor microenvironment and to model signaling pathway networks. Collectively, these algorithms are promoting the integration of multi-modal information in oncology, thereby accelerating the advancement of AI-driven precision medicine.

In endometrial cancer research and clinical applications, the aforementioned algorithms have all demonstrated promising performance. Convolutional neural networks, especially structures like ResNet, DenseNet, and U-Net, are widely used in imaging data processing tasks for endometrial cancer. They enable precise segmentation of endometrial tumors, assessment of the depth of myometrial invasion (44), and determination of cervical stromal involvement, thereby providing support for preoperative staging and risk stratification. In multi-omics data analysis, SVM and RF algorithms are commonly used to process high-dimensional data such as gene expression profiles, DNA methylation, and miRNA, and they possess strong classification and variable screening capabilities. SVM is effective in identifying molecular subtypes of endometrial cancer, while RF performs stably in feature importance assessment and risk prediction. Similarly, ensemble learning algorithms like XGBoost and LightGBM are often used to integrate clinical, pathological, and omics data to build prognostic models for endometrial cancer, predicting patient survival rates, recurrence risks, and treatment responses.

This study implemented several innovations in algorithm selection, model design, and task setup. Considering the complex glandular structures, diverse cell morphologies, and subtle inter-class differences in endometrial pathological images, we selected ResNet-18, a relatively lightweight architecture with strong generalization capability, as the backbone network. The ResNet architecture alleviates vanishing gradient and degradation problems in deep networks through its residual blocks. This design enables stable extraction of multi-level morphological features even with limited data, making it particularly suitable for medical image classification with finite samples. Compared to deeper networks such as ResNet-50 or ResNet-101, ResNet-18 maintains high feature extraction capability while having lower computational overhead, facilitating deployment in real clinical scenarios (45, 46). To further adapt to the multi-task learning system, this study implemented key improvements to the structural hierarchy and task strategy based on ResNet-18. By sharing lower-level convolutional layers, the model jointly learned discriminative features for EEC, hyperplastic lesions (typical and atypical), and physiological stages (proliferative, secretory, and menopausal), thereby improving feature reuse and avoiding redundant computation. Transfer learning strategies were introduced by loading pretrained parameters, strengthening the model’s feature extraction capability and training stability under the constraint of limited annotated samples. Secondly, in data preprocessing, a systematic image enhancement and filtering pipeline was constructed. Multi-channel filtering was used to optimize tissue region extraction, significantly reducing background interference and improving model input quality. Unlike traditional whole-slide image analysis methods, we employed a sliding-window approach combined with tissue density assessment to precisely locate high-information regions. This strategy dynamically filtered background areas, reducing ineffective computation and enhancing both efficiency and spatial representation capacity.

It is worth emphasizing that our study possessed a relatively ample number of high-quality samples for modeling endometrioid endometrial carcinoma, laying a solid foundation for model performance improvement and clinical application. We collected and included a total of 551 EEC slides confirmed by dual review from senior pathologists, of which over 460 samples were used for model training and validation. Compared to most existing studies with smaller sample sizes or single data sources, our data covered a broader range of morphological variations and tissue backgrounds, significantly improving the model’s coverage for the complex morphological spectrum of EEC. This enabled the model to achieve an F1-score of 0.95 and sensitivity of 96.06% on the internal test set, and particularly ensured accurate recognition of early micro-invasive foci and enhanced the model’s adaptability to real clinical scenarios. Large-sample training not only improved the model’s recognition accuracy for typical features but also enhanced its discriminative robustness for borderline cases and atypical hyperplasia, thereby providing strong data support for the subsequent promotion and application of the model in actual pathological diagnosis.

This study observed that the model exhibited near-perfect performance in the EEC identification task (F1-score 0.95), primarily attributable to hallmark changes in malignant cells such as increased nuclear-to-cytoplasmic ratio, nuclear pleomorphism, gland fusion, stromal invasion, and irregular cell morphology (47). These changes form highly specific morphological patterns in H&E-stained slides. In contrast, distinguishing atypical hyperplasia faced greater challenges (F1-score 0.74), because its transitional features with typical hyperplasia constituted the fundamental difficulty for algorithmic recognition. Notably, recognition performance was observed across physiological stages. The proliferative phase, characterized by typical straight tubular gland structures and pseudostratified epithelium (PPV 91.75%), significantly outperformed the secretory phase (PPV 80.94%). The latter exhibits subnuclear vacuolization in the early secretory phase and serrated glands in the late phase; this dynamic heterogeneity significantly increases recognition difficulty, accurately mirroring the core challenge in clinical pathological diagnosis.

**Figure 9:**
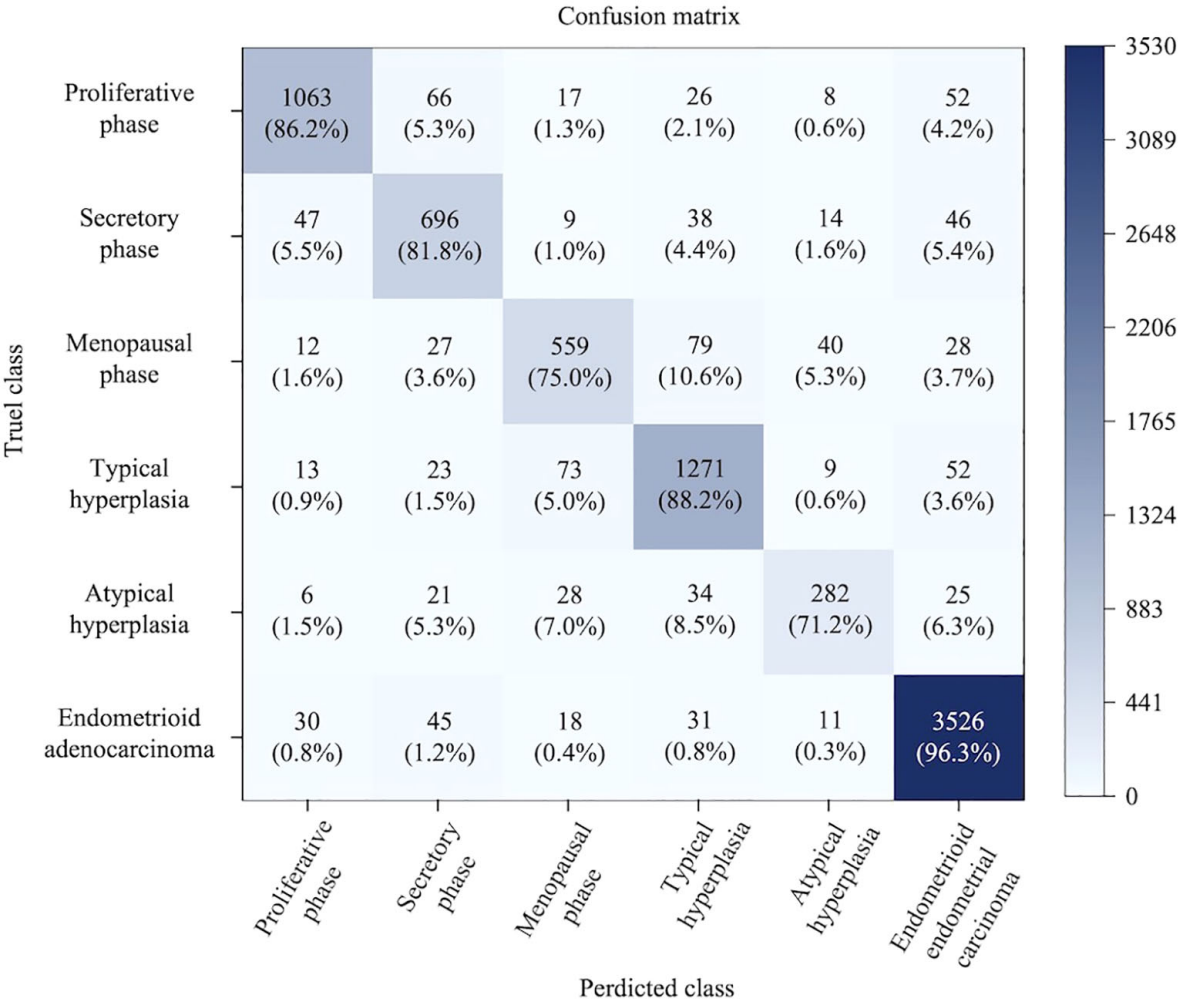
Six-class confusion matrix heatmap. This figure shows the classification performance of the multi-task deep learning model for endometrium on the test set. The model performs best at identifying endometrioid adenocarcinoma, while the identification of hyperplastic lesions and normal endometrial phases reaches clinically acceptable levels.

Quantitative analysis through the confusion matrix heatmap in the **Figure 9** revealed that key misclassification paths primarily occurred in (1): Secretory phase samples being misclassified as proliferative phase, mainly occurring in the late secretory phase where dilated glandular lumina resemble the pseudostratified epithelium morphology of the proliferative phase (48). (2): Approximately 5.3% of atypical hyperplasia samples were misclassified as secretory phase, because similar increases in glandular tortuosity and serrated structures also appear in the middle-late stages of atypical hyperplasia, and both are accompanied by stromal decidualization. (3): The low sensitivity of menopausal phase samples is considered closely associated with preparation artifacts; cleft-like structures and atrophic stroma in tissue sections are difficult to distinguish under low magnification. These phenomena reveal the dual nature of the deep learning model: On the one hand, Convolutional Neural Networks (CNN) can effectively capture valid relationships and changes among glands, stroma, and cells; on the other hand, their performance is still limited by the insufficient expression of cellular information inherent in H&E staining.

These misclassification patterns indicate that the physiological stages of normal endometrium exhibit considerable morphological diversity. The normal menstrual cycle includes the proliferative, secretory, and menopausal phases, and under certain conditions, the endometrium in each of these phases may potentially progress to endometrial carcinoma. During the proliferative phase, sustained estrogen stimulation without sufficient progesterone can lead to abnormal hyperplasia, increasing the risk of malignant transformation; the secretory phase generally carries a lower risk, but insufficient progesterone or continued estrogen exposure may allow lesions originating from proliferative-phase hyperplasia to further progress; in the menopausal phase, the endometrium is typically atrophic, but exogenous estrogen exposure or metabolic abnormalities such as obesity and diabetes can elevate the risk of malignancy. Therefore, even as normal tissue, all these physiological stages possess potential for malignant transformation. The model is capable of accurately identifying different physiological stages, and its criteria align with clinical pathological features. It primarily relies on key morphological characteristics for distinction, including stromal changes (dense or edematous, decidualized), glandular structures (straight tubular, serrated, dilated, or fragmented), and vascular alterations (spiral artery proliferation or dilation). This capability not only helps establish a reliable baseline of normal tissue morphology but also provides critical support for preoperative risk assessment, surgical planning, and tissue sampling decisions, enabling early identification of potentially high-risk backgrounds, optimizing diagnostic workflows, and reducing the risk of missed diagnoses.

The model demonstrated promising performance in identifying EEC, hyperplastic lesions, and normal physiological stages, confirming its clinical application value. Regrettably, the significant drop in cancer recognition rate on the independent validation set (84.86% vs 96.06%) suggests that the model still has some deficiencies. One issue lies in the generalizability of the trained model to external datasets, revealing single-center data bias. In this model, we only collected slides from the same hospital, failing to prove the model’s generalizability to other external data. Different datasets involve variations in H&E-stained tissue slide processing (including tissue sectioning, fixation materials, staining concentration, and other factors), ultimately leading to differences in slide quality (49, 50). This may cause overfitting: the model tends to memorize specific patterns and fails to generalize to new datasets, reducing accuracy and performance. Furthermore, the collected slides were predominantly large specimens confirmed by laparoscopy or laparotomy, resulting in specimen type imbalance. Small curettage specimens were not validated. Curettage may distort tissue morphology, and small specimens have limitations in reflecting tumor histomorphology. Therefore, models trained on large specimens may perform poorly on small specimens. More datasets should be collected from different institutions to ensure specimen diversity and enhance model applicability. Another shortcoming is that despite achieving 96.14% sensitivity for EEC detection, the deep learning model only reached preliminary diagnosis and failed to perform further subtyping (e.g., estrogen-dependent vs. estrogen-independent), for which adjuvant treatment strategies are completely different. If the deep learning model could achieve pathological subtype identification, it would provide higher-dimensional intelligent support for clinical treatment decisions. In the future, we will further optimize the model to achieve higher-level subtype discrimination, promoting its widespread application in real clinical environments.

In conclusion, we have established a reliable auxiliary diagnostic system for endometrioid endometrial carcinoma that simultaneously enables the identification of endometrial hyperplastic lesions and physiological phases of normal endometrium. We recognize that pathological nuances significantly influence model diagnostics, and the professional expertise and analytical experience of pathologists remain the cornerstone of technological advancement. AI models trained on well-curated and standardized datasets will facilitate the clinical implementation of AI pathology. We believe that in the near future, with the expansion and accumulation of datasets, the performance of deep learning models will substantially improve, enabling better integration with clinical practice and more precise analysis of endometrial tissues as well as deeper molecular-level investigations.

## Data Availability

The raw data supporting the conclusions of this article will be made available by the authors, without undue reservation.
